# Fetal Exposure to Environmental Neurotoxins in Taiwan

**DOI:** 10.1371/journal.pone.0109984

**Published:** 2014-10-09

**Authors:** Chuen-Bin Jiang, Hsing-Cheng Hsi, Chun-Hua Fan, Ling-Chu Chien

**Affiliations:** 1 Department of Pediatrics, Taipei Mackay Memorial Hospital, Taipei, Taiwan; 2 Mackay Junior College of Medicine, Nursing, and Management, Taipei, Taiwan; 3 Graduate Institute of Environmental Engineering, National Taiwan University, Taipei, Taiwan; 4 School of Public Health, Taipei Medical University, Taipei, Taiwan; The Ohio State University, United States of America

## Abstract

Mercury (Hg), lead (Pb), cadmium (Cd), and arsenic (As) are recognized neurotoxins in children that particularly affect neurodevelopment and intellectual performance. Based on the hypothesis that the fetal basis of adult disease is fetal toxic exposure that results in adverse outcomes in adulthood, we explored the concentrations of key neurotoxins (i.e., Hg, Pb, Cd, and As) in meconium to identify the risk factors associated with these concentrations. From January 2007 to December 2009, 545 mother-infant pairs were recruited. The geometric mean concentrations of Pb and As in the meconium of babies of foreign-born mothers (22.9 and 38.1 µg/kg dry weight, respectively) were significantly greater than those of babies of Taiwan-born mothers (17.5 and 33.0 µg/kg dry weight, respectively). Maternal age (≥30 y), maternal education, use of traditional Chinese herbs during pregnancy, and fish cutlet consumption (≥3 meals/wk) were risk factors associated with concentrations of key prenatal neurotoxins. The Taiwan government should focus more attention on providing intervention programs for immigrant mothers to help protect the health of unborn babies. Further investigation on how multiple neurotoxins influence prenatal neurodevelopment is warranted.

## Introduction

Childhood can be regarded as a sequence of life stages from conception through fetal development, infancy, and adolescence [1]. Vulnerable children may be exposed to various environmental toxicants during the stages of gestation, neonate, infant, and toddler. The Council on Environmental Health, the American Academy of Pediatrics (AAP) summarized several biomonitoring data from the Centers for Disease Control and other prevention biomonitoring programs in the United States and indicated that a variety of harmful chemicals, including metals, can be found in adults or child’s blood, cord blood, and breast milk [2]. The AAP is concerned with the particular vulnerabilities of children and pregnant women and advocates enforcing a metal management policy [2]. Maternal body burden and recent exposure are major toxic sources for the fetus, because toxins can pass through the placenta and blood-brain barrier into the fetal bloodstream. The hypothesis regarding the “fetal basis of adult disease” indicates that fetal toxic exposures may result in adverse outcomes in adulthood [3]–[5]. Heavy metals, such as mercury (Hg), lead (Pb), cadmium (Cd), and arsenic (As), are recognized neurotoxins in children that particularly affect neurodevelopment and intellectual performance [6]. An epidemiologic study in Shanghai attempted to determine the corresponding effects on prenatal exposure to multiple toxic heavy metals and the Neonatal Behavioral Neurological Assessments (NBNA) scores for children older than 2 years [7]. The study found that high Hg, Cd, and Thallium concentrations in cord blood were negatively associated with NBNA scores. Previous studies have reported that postnatal As exposure increased the risk of impaired cognitive development in childhood [8], [9]. Recent studies have indicated that Pb exposure in the first 3 years of life exhibited the most long-lasting and damaging effects because of a less developed blood-brain barrier that allowed Pb to pass into developing brains [10], [11]. Early Pb exposure has been documented to cause intrauterine growth and developmental retardation, thus influencing future intellectual performance [6], [12]–[14]. Toxic exposure in utero warrants concern because of the effects on fetal brain development. Three epidemiologic studies have focused on the effect of prenatal Hg exposure on the neural development of children. The geometric mean Hg concentrations in the Faroe Islands were 22.9 μg/L (n = 894; interquartile range [IQR], 13.4–41.3 μg/L) and 4.27 μg/g (n = 914; IQR, 2.6–7.7 μg/g) in cord blood and maternal hair, respectively [15]. A correlation existed between neurodevelopmental performance and Hg concentration in cord blood at birth. The mean Hg concentrations were 6.8±4.5 μg/g (n = 711) and 6.5±3.3 μg/g (n = 708) in maternal and child hair at 66 months, respectively, in Seychelles [16], [17]. A New Zealand cohort study found no significant associations between maternal hair Hg concentration during pregnancy and the psychological test performance of children, but provided support for a significant relationship between prenatal Hg exposure and neurodevelopment [18]. Trasande et al investigated the health and economic consequences of methyl Hg exposure attributed to Hg emitted from American power plants [19]. They found that each year in the United States, more than 316588 babies had cord blood Hg levels >5.8 µg/L, which can cause lifelong loss of intelligence. Because of intelligence loss, the amount of lost productivity was estimated to be US$8.7 billion each year. In summary, children exposed to Hg in the prenatal or postnatal period may have a reduction in neurobehavioral performance and alteration in psychomotor development.

Toxins can cross the placenta and reach the fetal side, and numerous toxins can be determined in maternal urine, cord blood, and meconium [20]. Thus, meconium is a useful indicator of fetal accumulation of environmental toxins [21], [22]–[26]. The lifestyle and nutritional status of mothers is recognized to affect the degree of exposure of their unborn children. Moreover, meconium can be acquired easily and is representative of the second and third trimesters of fetal exposure during gestation.

Recently, a 12.89% rise in babies born to immigrant mothers has been observed in Taiwan. Most immigrant mothers became pregnant within 2 years of their stay. A study in Taiwan found that blood Pb levels were slightly higher in immigrant women than in native women (2.23±1.63 vs 1.63±1.00 µg/dl; *P* = 0.04), and the blood Pb levels decreased after 5 years [27]. To date, few studies have examined whether a significant difference in Hg, Pb, Cd, and As body burden exists between babies of immigrant and native mothers in Taiwan, which could substantially affect the health of children. We explored and systematically compared the concentrations of key neurotoxins (i.e., Hg, Pb, Cd, and As) in meconium between babies of Taiwan-born and foreign-born mothers. We also identified risk factors (e.g., maternal age and educational level, Chinese herbs, and fish intake) associated with fetal accumulation of these key neurotoxins. The obtained results clarify whether immigrant or native maternal exposure had greater influence on a baby’s body burden.

## Methods

### Study Population

The study population comprised 545 mother-infant pairs (398 Taiwan-born mothers and 147 foreign-born mothers) living in Northern Taiwan who were recruited between January 2007 and December 2009. The mothers were recruited in the third trimester (after 24 weeks) at the outpatient clinic. They delivered their babies at medical centers in Northern Taiwan, either at Taipei Mackay Memorial Hospital or at its branch in Hsinchu. Although all of the collected mother**-**infant pairs had uncomplicated pregnancies and deliveries, only those with single live births were recruited. After delivery, the mothers were interviewed face-to-face by a trained interviewer. Information pertaining to each mother’s sociodemographic characteristics, occupation, pregnancy and reproductive history, fish intake, other lifestyle characteristics, and the baby’s sex, gestational age, birth weight, head circumference, and height at birth was collected by a structured questionnaire. In our previous study [28], we have recruited 198 mother-infant pairs residing in the city of Hsinchu, including 184 Taiwan-born mothers and 14 foreign-born mothers, and successfully elucidated the relationships between Hg concentrations in meconium and maternal daily Hg exposure dose. However, because the population of 14 foreign-born mother-infant pairs is small, the influences of the immigrant or native maternal exposure on the baby’s Hg body burden could not be successfully elucidated in that study [28]. To enhance understanding of the body burden of Hg, Pb, Cd, and As in babies of Taiwan-born and foreign-born mothers, we increased the sample size to 545 and subsequently evaluated the concentration of Hg, Pb, Cd, and As in meconium. Meconium specimens were collected from 545 healthy newborn infants.

This work was approved by the Institutional Review Board of Taipei Medical University (P940030) and Taipei Mackay Memorial Hospital (MMH-I-S-596). Written informed consents were prepared for both participating mothers and infants. All of the participating mothers and infants’ parents signed and returned the written informed consent for this research. All of the processes were done in accordance with the Declaration of Helsinki and relevant policies in Taiwan.

### Sample Collection and Metal Analysis

We collected meconium samples on the first postnatal day and froze them in a freezer at –20°C before analysis. Approximately 30 mg of the lyophilized meconium sample was transferred to a boat; activated alumina, sodium carbonate, and calcium hydroxide were subsequently added. Hg concentrations were measured using Hg cold-vapor atomic absorption spectroscopy (Nippon Instruments Mercury/MA-2000SC), and each sample was measured in triplicate. To determine the concentrations of Pb, Cd, and As, approximately 0.5 g of a preconditioned meconium sample was digested in flasks for 2 hours by using 5 mL of nitric acid at 100°C in a water bath. After cooling, the residual fluid was diluted to 10 mL with distilled water. The concentrations of Pb, Cd, and As were determined using an inductively coupled plasma/mass spectrometer (Thermo X-series II). The standard reference material, 1566b Oyster Tissue, was used to ensure the precision and accuracy of meconium analyses. The precision and accuracy were 91.9–96.7% and 97.9–99.8%, respectively. Recoveries of the spiked samples (n = 5) were 101±2%, 98±2%, 100±2%, and 100±2%, and the detection limits were 0.23 ng/g, 0.08 µg/L, 0.23 µg/L, and 0.12 µg/L for Hg, Pb, Cd, and As analyses, respectively.

### Statistical Analysis

The distributions of continuous variables were presented as mean ± standard deviation (SD). Hg, Pb, Cd, and As concentrations in meconium were presented as geometric mean due to the right-skewed distribution. Differences between groups in age, gestation, and weight were evaluated using the t-test, whereas the chi-square test was conducted to evaluate the independence of two categorical variables. When data were not normally distributed, the Wilcoxon rank sum test was employed to examine the body burden; namely, the differences in Hg, Pb, Cd, and As concentrations in meconium between babies of foreign-born and Taiwan-born mothers. The right-skewed data were normalized using logarithmic transformation for statistical analyses.

Multinomial regression was conducted to predict the effects of maternal characteristics on specific groups, compared with the reference group, to calculate adjusted odds ratios (aORs) and 95% confidence intervals (CIs). The babies were divided into four groups: (1) reference (first quartile); (2) Q2 (second quartile); (3) Q3 (third quartile); (4) Q4 (fourth quartile) based on the Hg, Pb, Cd, and As concentrations in meconium.

All statistical analyses were completed using SAS 9.1 software for Windows (SAS Institute Inc., Cary, NC, USA.) and *P*<0.05 was considered statistically significant.

## Results

Based on the demographic characteristics of the 545 mother-infant pairs (Table 1), the mean age of the Taiwan-born mothers (34.5 y) was significantly greater than that of the foreign-born mothers (31.3 y). The mean baby birth weights were significantly different between the babies of foreign-born (3178 g) and Taiwan-born mothers (3135 g). The mean length of gestation of the babies of foreign-born mothers (38.9 wk) was significantly longer than that of the babies of Taiwan-born mothers (38.6 wk), and 16.3% of the mothers consumed traditional Chinese herbs while pregnant. The consumption frequencies significantly differed between the foreign-born and the Taiwan-born mothers.

**Table 1:** Demographic characteristic of mother-infant pairs (n = 545).

Characteristic	All subjects(n = 545)	Taiwan-born(n = 398)	Foreign-born(n = 147)	*P* value
Age (years)	33.6(29.0–38.2)	34.5(30.2–38.8)	31.3(26.6–36.0)	<.0001
Maternal height (cm)	162(156–168)	163(156–170)	158(153–164)	0.005
Pre-pregnant weight (kg)	54(44–64)	56(46–66)	50(44–56)	<.0001
pregnant weight (kg)	68(59–78)	69(58–80)	65(58–72)	<.0001
Gestation (weeks)	38.7(37.3–40.1)	38.6(37.1–40.1)	38.9(37.6–40.2)	0.01
Head circumference (cm)	33.4(32.1–34.8)	33.3(32.0–34.6)	33.5(31.8–35.2)	0.24
Baby’s height (cm)	49.7(46.2–53.2)	49.6(45.6–53.6)	50.0(47.3–52.7)	0.74
Baby’s birth weight (g)	3147(2685–3609)	3135(2687–3585)	3179(2680–3678)	0.03
Sex of newborn baby				0.88
Male	274(50.6)	202(50.8)	72(50)	
Female	268(49.4)	196(49.2)	72(50)	
Parity				0.46
≤1	318(58.3)	236(59.3)	82(55.8)	
≥2	227(41.7)	162(40.7)	65(44.2)	
Occupational exposure				0.1
Yes	52(9.5)	43(10.8)	9(6.1)	
No	493(90.5)	355(89.2)	138(93.9)	
Amalgam fillings during pregnancy				0.58
Yes	17(3.2)	14(3.6)	3(2.1)	
No	514(96.8)	373(96.4)	141(97.9)	
Smoking during pregnancy				0.11
Yes	8(1.5)	8(2.1)	0(0)	
No	516(98.5)	371(97.9)	145(100)	
Alcohol consumption				0.33
Yes	111(34.7)	65(32.7)	46(38)	
No	209(65.3)	134(67.3)	75(62)	
Consumed traditional Chinese herbs				0.01
Yes	89(16.3)	75(18.8)	14(9.5)	
No	456(83.7)	323(81.2)	133(90.5)	
Fish cutlet consumption				0.1
<1 meal/week	142(31)	111(32.6)	31(26.5)	
1–2 meals/week	197(43)	150(44)	47(40.2)	
≥3 meals/week	119(26)	80(23.4)	39(33.3)	
Sashimi consumption				0.38
<1 meal/week	223(94.9)	182(95.8)	41(91)	
1–2 meals/week	11(4.7)	7(3.7)	4(9)	
≥3 meals/week	1(0.4)	1(0.5)	0(0)	

**Figure 1:**
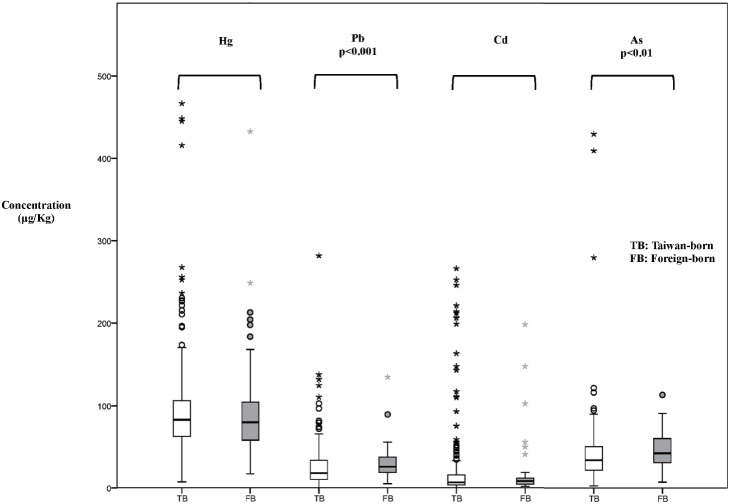
Box and whisker plots display distributions of Hg, Pb, Cd, and As concentrations in meconium (µg/kg) among babies of Taiwan-born and foreign-born mothers. Median (horizontal line in the box), minimum, and maximum are shown. The box includes 50% of values and is limited by the 25^th^ and 75^th^ percentiles.

The box and whisker plots in Figure 1 display the distributions of Hg, Pb, Cd, and As concentrations in meconium among the babies of Taiwan-born and foreign-born mothers. The Hg and Cd concentrations in meconium were not significantly different between the 2 groups. The geometric mean concentrations of Hg and Cd in meconium in Taiwan were 79.1 and 8.71 µg/kg dry weight, respectively. The geometric mean concentrations of Pb and As in meconium of the babies of foreign-born mothers (22.9 vs 38.1 µg/kg dry weight) were significantly greater than those of the babies of Taiwan-born mothers (17.5 vs 33.0 µg/kg dry weight). Because of the observed significant difference between the babies of foreign-born and Taiwan-born mothers, we further estimated the risk factors of Pb concentration in meconium by using multiple linear regressions. Table 2 shows that Pb concentration in meconium was associated with maternal nativity, the baby’s birth weight, use of traditional Chinese herbs during pregnancy, and As concentration in meconium. As and Pb concentrations in meconium had a high correlation (r = 0.47, *P*<0.05).

**Table 2:** Multiple linear regression model for meconium Pb concentration.

Variable in model	β	*P* value
Nativity^a^	0.32	0.03
Age	0.0006	0.97
Body weight	−0.0004	0.01
Parity	0.23	0.36
Consumed traditional Chinese herbs	0.37	0.01
As concentration in meconium	0.65	0.0001

a Foreign-born mothers vs. Taiwan-born mothers.

The independent association of maternal characteristics with specific groups (Q2 through Q4) compared with the reference group was assessed using multinomial logistic regression (Table 3). Each variable in the model was adjusted for the remaining variables. The highest risk for the Q4 Hg group compared with the reference was higher maternal educational attainment (high school aOR: 2.89; 95% CI: 1.08–7.75 vs college or higher aOR: 2.70; 95% CI: 1.08–6.75) and fish cutlet consumption ≥3 meals/week (aOR: 2.89; 95% CI: 1.45–5.77). The Q4 Pb group was independently associated with the use of traditional Chinese herbs during pregnancy (aOR: 1.95; 95% CI: 1.08–3.51) and fish cutlet consumption 1–2 meals/week (aOR: 1.87; 95% CI: 1.01–3.51). The high risk for the Q4 Cd group compared with the reference was maternal age ≥30 years (aOR: 2.49; 95% CI: 1.01–6.11), which was similar to that of the Q3 Cd group (aOR: 2.40; 95% CI: 1.03–5.61). The Q4 As body burden was due to older maternal age (≥30 y, aOR: 2.67; 95% CI: 1.08–6.58) and the highest fish cutlet consumption (≥3 meals/week, aOR: 2.31; 95% CI: 1.31–5.23).

**Table 3:** Multinomial regression model assessing characteristics by Hg, Pb, Cd, and As meconium levels.

Variable	Hg						Pb						Cd						As					
	Q2		Q3		Q4		Q2		Q3		Q4		Q2		Q3		Q4		Q2		Q3		Q4	
	OR	95% CI	OR	95% CI	OR	95% CI	OR	95% CI	OR	95% CI	OR	95% CI	OR	95% CI	OR	95% CI	OR	95% CI	OR	95% CI	OR	95% CI	OR	95% CI
Maternal age (yrs)																								
<30	Ref.		Ref.		Ref.		Ref.		Ref.		Ref.		Ref.		Ref.		Ref.		Ref.		Ref.		Ref.	
≧30	1.09	0.58–2.06	1.59	0.80–3.14	1.12	0.59–2.14	0.96	0.48–1.92	2.67	1.08–6.59	2.33	0.94–5.77	1.59	0.70–3.59	2.4	1.03–5.61	2.49	1.01–6.11	1.63	0.75–3.55	1.6	0.71–3.60	2.67	1.08–6.58
Educational status																								
Less thanhigh school	Ref.		Ref.		Ref.		Ref.		Ref.		Ref.		Ref.		Ref.		Ref.		Ref.		Ref.		Ref.	
High School	1.71	0.71–4.10	1.97	0.80–4.86	2.89	1.08–7.75	3.25	0.71–15.0	0.65	0.25–1.66	0.56	0.20–1.61	0.64	0.20–2.10	0.9	0.34–2.41	0.85	0.27–2.65	1.36	0.42–4.40	0.45	0.15–1.32	0.61	0.23–1.65
College or more	1.67	0.76–3.69	1.71	0.75–3.90	2.7	1.08–6.75	4.17	0.96–18.2	0.61	0.26–1.41	0.86	0.35–2.13	1.53	0.55–4.21	0.82	0.33–2.02	1.54	0.56–4.20	1.63	0.54–4.92	0.98	0.40–2.41	0.79	0.33–1.89
Occupationalexposure																								
No	Ref.		Ref.		Ref.		Ref.		Ref.		Ref.		Ref.		Ref.		Ref.		Ref.		Ref.		Ref.	
Yes	0.92	0.34–2.52	1.68	0.69–4.09	1.87	0.77–4.52	1.05	0.38–2.87	1.21	0.47–3.12	1.53	0.62–3.77	1.72	0.73–4.09	0.35	0.08–1.53	1.12	0.44–2.87	0.58	0.17–1.98	1.99	0.87–4.58	1.19	0.46–3.07
TraditionalChineses Herbs																								
No	Ref.		Ref.		Ref.		Ref.		Ref.		Ref.		Ref.		Ref.		Ref.		Ref.		Ref.		Ref.	
Yes	1.41	0.87–2.29	1.61	0.98–2.63	1.63	0.99–2.68	1.04	0.61–1.78	1.19	0.69–2.09	1.95	1.08–3.51	1.95	1.08–3.51	1.19	0.68–2.06	1.18	0.68–2.03	1.56	0.90–2.72	1.86	1.04–3.32	0.94	0.54–1.63
Fish cutletconsumption																								
<1 meal/week	Ref.		Ref.		Ref.		Ref.		Ref.		Ref.		Ref.		Ref.		Ref.		Ref.		Ref.		Ref.	
1–2 meal/week	1.11	0.65–1.89	1.34	0.78–2.29	1.37	0.80–2.37	1.38	0.76–2.50	1.06	0.56–2.00	1.87	1.01–3.51	2.04	1.07–3.87	1.15	0.62–2.14	1.45	0.79–2.66	1.31	0.72–2.38	1.05	0.57–1.93	1.21	0.63–2.32
≧3 meal/week	2.27	1.14–4.50	2.42	1.20–4.87	2.89	1.45–5.77	1.14	0.54–2.40	1.79	0.90–3.57	1.6	0.74–3.46	1.82	0.84–3.92	1.34	0.65–2.76	1.42	0.69–2.95	1.48	0.71–3.08	1.3	0.62–2.72	2.61	1.31–5.23

Note:

a aOR: adjusted odds ratio for parity, gestation (weeks) and with variables run simultaneously; Hg, Pb, Cd and As meconium levels below the first quartile are the reference groups.

As (µg/kg): Q1: 0–23.5 (reference); Q2: 23.6–36.6; Q3: 36.7–52.0; Q4: >52.1.

Cd (µg/kg): Q1: 0–3.7 (reference); Q2: 3.8–7.3; Q3: 7.4–14.9; Q4: >15.0.

Pb (µg/kg): Q1: 0–11.2 (reference); Q2: 11.3–19.4; Q3: 19.5–34.9; Q4: >35.0.

Hg (µg/kg): Q1: 0–62.2 (reference); Q2: 62.3–82.6; Q3: 82.7–105.8; Q4: >105.9.

## Discussion

This study provides essential information regarding in utero exposure to key neurotoxins (Hg, Pb, Cd, and As), as well as key risk factors, such as maternal age (≥30 y), maternal education, use of traditional Chinese herbs during pregnancy, and fish cutlet consumption (≥3 meals/wk), that are associated with concentrations of fetal neurotoxin body burden. In addition, immigrant mothers have greater influences than those of Taiwan-born mothers on the baby’s body burden, particularly on Pb and As.

The Hg, Pb, Cd, and As concentrations in meconium in this study were comparable to those found in other studies (Table 4) [21], [30], [31]. The median concentration of Hg in meconium in Taiwan was 82.6 µg/kg dry weight. The Hg concentration in this study was much greater than those in other studies conducted in the Philippines and Austria. The difference in meconium Hg concentration among countries may stem from the differences in fish intake amount and frequency. The median concentration of Pb in meconium in Taiwan was 19.4 µg/kg dry weight. The Pb concentration in this study was much lower than those in other studies in performed in Manila, the Philippines (with a high environmental pollution index) and Kocaeli, Turkey (an industrial center).

**Table 4:** Comparison of Hg, Pb, Cd, and As concentrations (µg/kg) in meconium in different studies.

	PositivePercentage	Median	Range/IQR^a^	Reference
Manila, Philippines (n = 426)				Ostrea Jr. et al., 2002
Hg (µg/kg)	83.9	3.17	0.43–71.6	
Pb (mg/kg)	26.5	35.8	8.23–603	
Cd (mg/kg)	8.50	13.4	2.09–27039	
Kocaeli, Turkey (n = 117)				Türker et al., 2006
Pb (mg/kg)	100	46.5	1399^a^	
Cd (mg/kg)	100	2.3	55.6^a^	
Vienna, Austria(n = 36)				Gundacker et al., 2010
Hg (µg/kg)	100	4.00	0.40–128	
Pb (µg/kg)	100	15.5	1.90–103	
Northern Taiwan(n = 545)				This study
Hg (µg/kg)	100	82.6	43.9^a^	
Pb (µg/kg)	100	19.4	24.0^a^	
Cd (µg/kg)	100	7.27	10.9^a^	
As (µg/kg)	100	37.3	28.6^a^	

a IQR, Inter-quartile range: third quartile to first quartile.

The geometric mean level of Pb in the meconium of the babies of foreign-born mothers (22.9 µg/kg dry weight) was significantly higher than that of the babies of Taiwan-born mothers (17.5 µg/kg dry weight). The results are consistent with the findings of Wu et al [27], that immigrant married women (from Vietnam, Mainland China, and other Southeast Asian countries) had greater mean blood Pb concentrations than those of native Taiwanese women (2.23±1.63 vs 1.63±1.00 µg/dL; *P* = 0.04). The blood Pb concentration was negatively correlated with the years of migration among immigrant married women (r = 0.254; *P* = 0.03) [27]. A possible explanation is that immigrant married women in our study had low education and low income levels compared with women born in Taiwan. The socioeconomic status may influence their living environment, dietary pattern, and health habits. Thus, the poor socioeconomic status may influence their blood Pb levels. The results substantially agree with earlier findings, that blood Pb levels were positively correlated with recent immigration to the United States, poor socioeconomic status, and low education levels in pregnant women [29].

In our study, babies of mothers who consumed traditional Chinese herbs during pregnancy had an increased meconium Pb level. Regarding traditional Chinese herbs used in Taiwan, approximately 90% of mothers use medicinal powders and decoctions of medicinal ingredients. Approximately 40% use herbal remedies to maintain optimal health and 35% choose therapy against disease during pregnancy. Maternal blood Pb levels are associated with increased risk of spontaneous abortion, preterm births, and other anomalies [32]–[34]. Maternal blood Pb levels can be influenced by environmental exposure, lifestyle, and nutritional status, and can affect the fetus by crossing the placenta. Our previous study indicated that the breast milk Pb level in the consumption group (mothers who consumed traditional Chinese herbs) was 8.59±10.95 µg/L, significantly greater than that of the control group (mothers who did not consume traditional Chinese herbs; 6.84±2.68 µg/L) [35]. An Austrian study examining meconium and maternal blood Pb levels (median 15.5 µg/kg dry weight vs 2.5 µg/dL) suggested that even low Pb exposure in utero can influence birth length and weight [30]. Our study showed that meconium Pb level was negatively associated with birth weight (Table 2). Certain studies have provided convincing evidence that Pb exposure in early life can affect children’s physical and mental development in later life [36]. This should be a concern for protecting infants and for further follow-up regarding adverse health outcomes.

The results presented here suggest that Hg concentrations in meconium were not significantly different between the babies of Taiwan-born and foreign-born mothers (Figure 1). The frequency of fish intake was the key factor influencing Hg concentration in mothers and their babies. These results are consistent with those of our previous studies [28], that mothers who consumed fish more frequently had greater blood Hg concentrations. Additionally, 89% of the mothers had a blood level greater than the U.S. National Research Council recommended value of 5.8 µg/L, which could cause the meconium Hg concentration to reach statistical significance [28], [37]. Based on results of our previous study from the Monte Carlo simulation, the crucial factors include Hg concentration in fish and the ingestion rate of fish. The values found in pregnant women in Taiwan are worrisome [37]. Asian women and women with higher income levels were found to eat more fish and had higher blood Hg in the National Health and Nutrition Examination Survey [38]. We observed a similar pattern in this study, wherein the highest aORs among the Q4 Hg group versus the reference group are for fish cutlet consumption ≥3 meals/week (2.89; 95% CI: 1.45–5.77) and maternal educational attainment (high school, 2.89 and college or higher, 2.70).

A birth cohort study recruited 289 pairs of mothers and infants in Taiwan, and the mean Cd level was 1.15 µg/L for maternal blood and 0.67 µg/L for cord blood. Prenatal Cd exposure may have a detrimental effect on head circumference at birth, and height, weight, and head circumference in the first 3 years of life [39]. Tian et al evaluated cord blood Cd level at birth and intelligence quotient (IQ) development at 4.5 years in 106 children in Da-Ye County, Hubei Province in Central China. They found that infants with higher cord blood Cd level had a significantly higher frequency in low birth weight (<2500 g), which influenced IQ development at 4.5 years [40]. We found no association between fetal growth and meconium Cd level (median 7.27 µg/kg dry weight). The inconsistency may be because of lacking information on the relationship between Cd levels in maternal or cord blood and meconium.

The current findings indicate that any increase in meconium As level by 1 µg/kg dry weight could reduce head circumference by 1.22 mm (data not shown). A previous study demonstrated that mothers with high levels of As exposure during pregnancy had a 6-fold increased risk of stillbirth [41]. Rahman et al suggested that mothers with low level As exposure (urinary arsenic concentration <100 µg/L) may have an effect on birth size [42]. When the urinary level increased by 1 µg/L, the corresponding birth weight, head circumference, and chest circumference reduced by 1.68 g, 0.05 mm, and 0.14 mm, respectively. Despite the paucity of relevant studies on children, studies have indicated that As may cross the placenta, increase the risk of fetal death, and impair growth [43], [44].

Our study has several strengths. Numerous mother-infant pairs were recruited for examining the concentrations of key neurotoxins in meconium, thus showing the accumulated exposure during fetal life. Exposure to multiple chemicals that act on the same adverse outcome can have a greater effect than exposure to an individual chemical, suggesting that exposure assessment of multiple chemicals is critical [45], [46]. Moreover, the babies of foreign-born mothers had greater meconium Pb and As levels than the babies of Taiwan-born mothers. The Taiwan government should focus more attention on providing intervention programs for immigrant mothers to help protect them and the health of their unborn babies.

This study also has some limitations. This is a single-center study covering only a limited sampling area in Northern Taiwan. Potential uncontrolled confounding effects, such as maternal nutritional status and the demographic characteristic of fathers, have not been considered.

In conclusion, this study provides baseline data (i.e., Hg, Pb, Cd, and As levels) regarding prenatal exposure. We demonstrated that maternal age (≥30 y), maternal educational attainment, use of traditional Chinese herbs during pregnancy, and fish cutlet consumption (≥3 meals/wk) are risk factors that can influence the concentrations of key neurotoxins in meconium and therefore, the fetus. Maternal nativity was shown to be a crucial factor for their baby’s Pb body burden. Further study is warranted to provide insights on how multiple neurotoxins influence later neurodevelopment.
